# Integrated Molecular Characterization to Reveal the Association between Kynurenine 3-Monooxygenase Expression and Tumorigenesis in Human Breast Cancers

**DOI:** 10.3390/jpm11100948

**Published:** 2021-09-24

**Authors:** Yuk-Wah Tsang, Chi-Hsun Liao, Chiao-Hsu Ke, Chi-Wen Tu, Chen-Si Lin

**Affiliations:** 1 Department of Radiation Oncology, Ditmanson Medical Foundation Chia-Yi Christian Hospital, Chia-Yi City 600556, Taiwan; 07130@cych.org.tw; 2 Department of Biomedical Engineering, Chung Yuan Christian University, Taoyuan City 320314, Taiwan; 3 Department of Veterinary Medicine, School of Veterinary Medicine, National Taiwan University, Taipei 106216, Taiwan; r07629008@ntu.edu.tw (C.-H.L.); f08629002@ntu.edu.tw (C.-H.K.); 4 Department of Surgery, Ditmanson Medical Foundation Chia-Yi Christian Hospital, Chia-Yi City 600556, Taiwan; 00780@cych.org.tw

**Keywords:** breast cancer, KMO, bioinformatics, inflammation, biomarker

## Abstract

Kynurenine 3-monooxygenase (KMO) is overexpressed in several tumors and participates in the progression of breast cancer tumorigenesis, including cancer types such as triple-negative breast cancer (TNBC). This malignant gene is an enzyme in the kynurenine pathway, which is involved in the carcinogenesis of cancer through immune function manipulation. However, it remains unclear whether the role of the KMO contributes to tumorigenesis and immune functions in human breast cancer. In this study, we found that KMO was highly expressed in different types of tumors, especially in invasive ductal breast carcinoma. In addition, KMO expression was positively correlated with the malignant clinical features of patients with breast cancer, such as TNBC and a nodal-positive status, along with patients with a higher Nottingham prognostic index (NPI). Furthermore, the top ten KMO-correlated genes were the chemokines and pro-inflammatory cytokines known to be involved in the progression of various cancers, therefore, KMO may facilitate breast cancers via synergistically regulating inflammatory responses in tumors with these hub genes. Taken together, these findings highlight the tumor-promotion role of KMO in breast cancers and suggest that KMO can serve as a biomarker for prognosis prediction in breast cancer patients.

## 1. Introduction

Breast cancer (BC) is one of the most commonly diagnosed cancers and is the principal cause of cancer-related mortality in women worldwide [1]. These tumors are highly heterogeneous and can be divided into several subgroups. The categorization of breast cancer is determined by the expression of three membrane receptors, known as estrogen receptors (ER), progesterone receptors (PR), and human epidermal growth factor receptors type 2 (HER2). Based on these three receptors, Luminal A/B (ER+), HER2+, and triple-negative breast cancers (TNBC, ER-/PR-/HER2-) are identified [2] with different molecular profiles, clinical outcomes, and treatment responses [3]. Luminal type breast cancer is usually considered to be low-grade and shows good responses to hormone therapy [4]. HER2+ breast cancer usually grows aggressively, and HER2-targeted antibody therapy using pertuzumab/trastuzumab plus a taxane has been widely used as the first-line standard therapy in HER2-enriched BC patients [5]. TNBC accounts for 15–20% of all breast cancers; it is considered to be one of the most drug-resistant cancer types and is characterized by a high malignancy [6]. Due to the absence of molecular targets, no desirable targeted therapeutic agents have been clinically approved for TNBC, so the mortality rate remains high [7,8]. Furthermore, once TNBC metastasizes to a distal organ, the 5-year survival rate dramatically decreases to 45% from 76% [9]. The lack of targeted therapies and poor prognosis in TNBC indicates that the tumorigenicity of this subtype of breast cancer remains unclear. Therefore, uncovering the potential tumorigenesis and hub molecules that drive the malignancy of breast cancers are critical issues, and such investigations may reveal novel biomarkers that can lead to benefits for patients with breast cancer.

Our previous studies reported that kynurenine 3-monooxygenase (KMO) can affect cancer-stemness [10] and is overexpressed in several TNBC cells [11], which indicates that KMO plays a vital role in breast cancers. KMO is involved in the kynurenine pathway, where it catalyzes the hydroxylation of L-kynurenine (KYN) to 3-hydroxykynurenine (3-HK) and further regulates the downstream production of quinolinic acid (QA). Therefore, elevated KMO shifts the pathway towards the formation of 3-HK and QA and regulates the immune response and tumor tolerance [12,13]. On the other hand, a high level of QA can stimulate N-methyl-D-aspartate (NMDA) receptors, leading to tumor proliferation through the ERK pathway. NMDA receptors are known to initiate gene activation and cell proliferation and to promote cell survival via the extracellular signal-regulated kinase (ERK1/2) pathways [14]. Furthermore, the upregulation of the KMO gene in human hepatocellular carcinoma (HCC) and breast cancers is associated with a worse prognosis, as an elevated expression of KMO participates in the proliferation, migration, and invasion of tumors [15,16].

Transcriptomic analysis using high-throughput RNA sequencing (RNA-Seq) has been a powerful method used to probe gene behaviors and identifying biomarkers for cancer diagnosis [17,18]. Furthermore, RNA-Seq uncovers the genetic mechanisms underlying human diseases [19]. For example, the Cancer Genome Atlas (TCGA) has quantified the gene expression levels in 9736 samples taken from 33 cancer types, in addition to data taken from 726 adjacent normal tissue samples [20]. The Genotype-Tissue Expression (GTEx) project produced RNA-Seq data for over 9000 samples across 53 tissues from 544 healthy individuals [19,20]. These databases were compiled to decode several complex diseases using tens of thousands of cancerous and non-cancerous samples, providing integrated biological information of BC compared to normal tissues. Although genetic studies can help with understanding the functions of tumor-related genes and the roles of tumor cell signal pathways, most studies have focused on the discovery of differentially expressed genes (DEGs) [21]. Comprehensively understanding the high degree of interconnectivity among the DEGs may be functionally related to understanding other genes with similar expression patterns [22]. 

Though evidence related to the tumorigenic role of KMO has begun to be disclosed, comprehensive large-scale clinical analyses are needed to further demonstrate how KMO contributes to cancer development among co-expressed genes. In the present study, we retrieved gene expression patterns and clinical data from breast cancer patients using the TCGA and the GTEx project. The relationship between KMO and cancer establishment was further analyzed using bioinformatics functional tools, including the Gene Ontology (GO), the Kyoto Encyclopedia of Genes and Genomes (KEGG), and the Protein–Protein Interaction (PPI) databases, to probe whether the expression of KMO is correlated to tumorigenesis in breast cancers and thereby whether it could serve as a biomarker related to cancer malignancy. 

## 2. Materials and Methods

### 2.1. Oncomine Database Analysis for KMO Expression

The Oncomine database (https://www.oncomine.org; accessed on 5 May 2021) [23] is an online cancer microarray database that provides genome-wide expression analysis. It currently contains 715 datasets and 86,733 samples. The mRNA expression of KMO between cancer tissues and non-cancer tissues was compared via the Oncomine database. The search parameters and thresholds taht were used to find the datasets were set as follows: analysis type, cancer vs. normal analysis; data type, mRNA; *p*-value < 0.05; fold change > 1.5; gene rank, top 10%.

### 2.2. Data Mining in Gene Expression Profiling Interactive Analysis (GEPIA)

The GEPIA database (http://gepia.cancer-pku.cn; accessed on 5 May 2021) [20] is an online analysis database of RNA sequencing expression data. It contains data for 9736 tumors and 8587 normal samples from the Genotype-Tissue Expression (GTEx) and the Cancer Genome Atlas (TCGA) projects. The parameters and thresholds used to find the box plots were set as follows: Datasets selection, BRCA; |log2FC| cutoff, 1; *p*-value cutoff, 0.01; matched normal data, match TCGA normal and GTEx data.

### 2.3. cBioPortal Database Analysis

The cBioPortal (https://www.cbioportal.org; accessed on 5 May 2021) [24,25] is an open-access resource for exploring multidimensional cancer genomics datasets. The portal provides data on mutations, DNA copy numbers, mRNA expression, protein and phosphoprotein levels, DNA methylation, and unknown clinical data from the TCGA and TARGET cohorts, as well as from publications from various laboratories. The cBioPortal was used to investigate the KMO somatic mutations of breast cancers in the TCGA database (Firehose Legacy) and the genes correlated with KMO.

### 2.4. Breast Cancer Gene-Expression Miner v4.4 (bc-GenExMiner v4.4)

The bc-GenExMiner v4.4 (http://bcgenex.centregauducheau.fr; accessed on 5 May 2021 ) [26,27] is a statistical mining tool recording annotated published breast cancer transcriptomic data, and includes 54 annotated genomic datasets and the data for 10,001 patients with breast cancer. The bc-GenExMiner v4.4 was used to analyze the association between KMO expression and intrinsic molecular structures to assess its prognostic impact in human breast cancer.

### 2.5. Kaplan–Meier plotter

The Kaplan–Meier (KM) plotter (https://kmplot.com/; accessed on 5 May 2021) [28] can assess the prognostic significance of 54k genes from 21 cancer types. It was used to analyze the association between the KMO mRNA expression and survival rates, including overall survival (OS) and relapse-free survival (RFS) rates in patients with breast cancer. Samples taken from patients with breast cancer were divided into two groups based on the trichotomization of the KMO expression (T1 vs. T3). The log-rank *p*-value and a hazard ratio with 95% confidence intervals were calculated after comparing the two groups with a Kaplan–Meier survival plot.

### 2.6. University of California Santa Cruz (UCSC) Cancer Genomics Analysis

The UCSC Xena (http://xena.ucsc.edu/; accessed on 5 May 2021) [29] is an analysis platform that can be used to explore and visualize the functional genomics from many cancer genomics datasets, such as the TCGA, GTEx, and ICGC. The UCSC Xena was used to verify the hierarchical clustering of hub genes correlated with KMO.

### 2.7. PPI Network Construction and Hub Genes Screening

The cBioPortal and R2 [30] databases were used to find the top 200 correlated genes of KMO in breast cancer (BRCA, TCGA, respectively). Next, the Search Tool for the Retrieval of Interacting Genes (STRING) database (http://string-db.org/; accessed on 5 May 2021) [31] was applied to create a protein–protein interaction network of the intersecting genes across the two databases and the important modules in the PPI network were analyzed using the Cytoscape plugin, Molecular Complex Detection (MCODE) [32]. The parameters were set as a degree cutoff = 2, node score cutoff = 0.2, k-core = 2, and a maximum depth = 100. The Cytoscape plugin, cytoHubba [33], was also used to identify the top 10 hub genes of the PPI network using the Maximal Clique Centrality (MCC) method.

### 2.8. Statistical Analysis

The Student’s *t*-test was used to compare the KMO mRNA expression between BC tissue and non-cancerous breast tissue samples in the Oncomine database. In the GEPIA, the differential expression analysis of disease status (tumor or normal) was performed using one-way ANOVA analysis. The Dunnett–Tukey–Kramer test and Welch’s analysis were performed to compare the differences among groups in the Breast Cancer Gene-Expression Miner v4.4. The log-rank test was used to analyze the association between KMO mRNA expression and survival rates in the Kaplan–Meier (KM) plotter. Spearman’s correlation was used to evaluate the correlation of gene expression in the cBioPortal databases, and Pearson’s correlation was used for the R2 databases. Graphpad Prism 8 software was used to carry out statistical analyses. The data are expressed as mean ± SEM. Statistical significance was achieved when *p* value < 0.05.

## 3. Results

### 3.1. Transcription Level of KMO Is Significantly Upregulated in Human Breast Cancer

**Figure 1:**
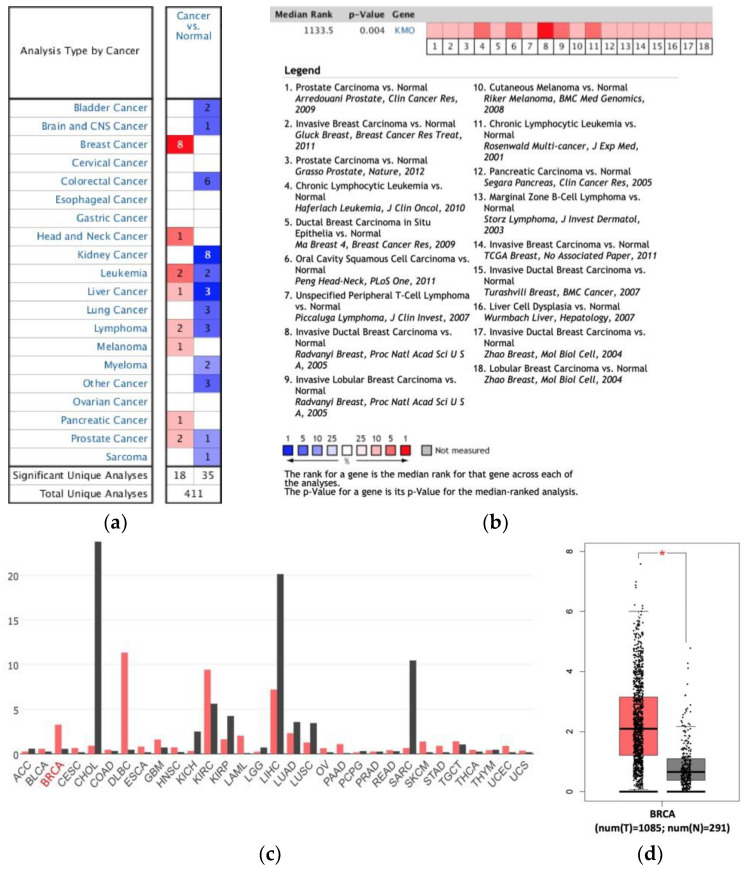
The transcription levels of KMO in human breast cancer. (**a**) The number of datasets with statistically significant KMO mRNA (cancer vs. corresponding normal tissue) overexpression (red) or downregulation (blue) in different types of cancers generated by Oncomine; (**b**) comparison of KMO overexpression across 18 datasets; (**c**,**d**) GEPIA database results revealing that compared with normal breast tissues (*n* = 291), the expression of KMO in breast cancer tissue (BRCA) was significantly upregulated (*n* = 1085) (*, *p* < 0.05).

**Figure 2:**
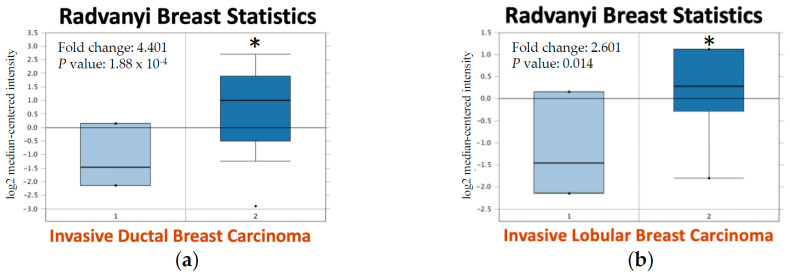

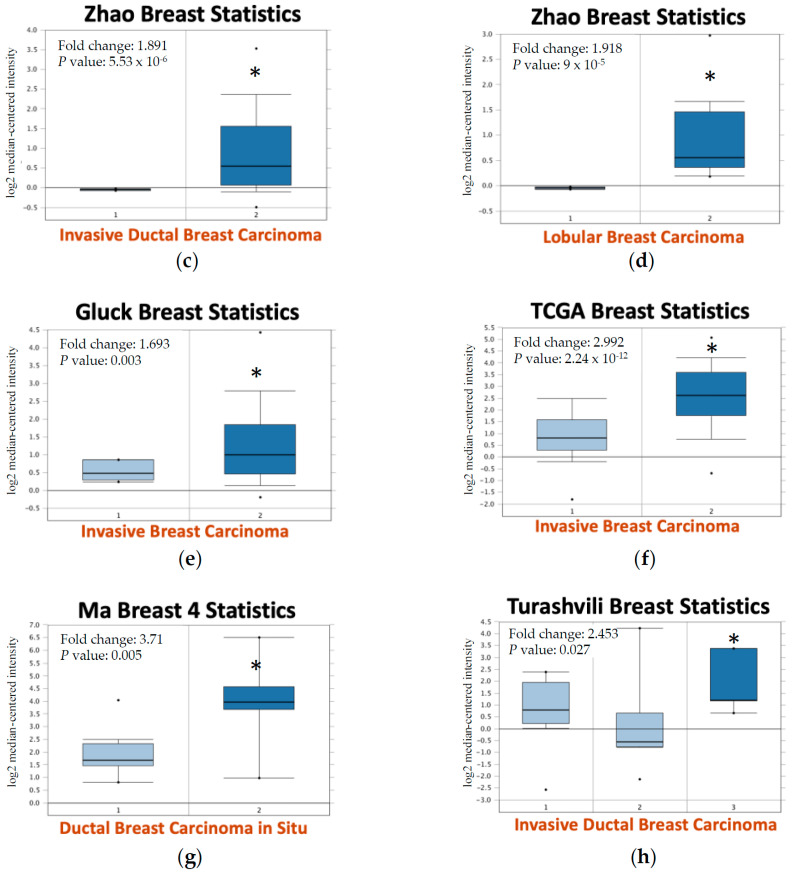
The analysis of KMO mRNA expression comparison between normal tissues and cancer tissues using the Oncomine database. (**a**,**b**) Analyzed from the ‘Radvanyi Breast Statistics’ cohort; (**c**,**d**) analyzed from the ‘Zhao Breast Statistics’ cohort; (**e**) analyzed from the ‘Gluck Breast Statistics’ cohort; (**f**) analyzed from the ‘TCGA Breast Statistics’ cohort; (**g**) Analyzed from the ‘Ma Breast 4 Statistics’ cohort; (**h**) analyzed from the ‘Turashvili Breast Statistics’ cohort. Statistical analysis was compared with related non-cancerous tissues. (1, non-cancerous tissues (**a**–**g**); 2, breast cancer tissues (**a**–**g**); 1, ductal breast tissues (**h**); 2, lobular breast tissues (**h**); 3, breast cancer tissues (**g**); *, *p* < 0.05).

The mRNA expression of KMO in various tumor samples was analyzed using the Oncomine database. KMO was significantly upregulated in eight types of cancers, and especially in breast cancers (Figure 1a). Among all 18 analysis datasets, almost half of the datasets (8/18) suggested that KMO was overexpressed in breast cancers (Figure 1b), which indicated that KMO was highly associated with breast cancer as compared with other types of cancers. Therefore, we further compared the levels of KMO mRNA expression in breast cancers (*n* = 1085) with those in normal breast tissues (*n* = 291) using the GEPIA database (Figure 1c,d), and the results also showed that KMO was overexpressed in breast cancers. To verify the roles of KMO in breast cancers, we validated the KMO expression in different types of breast cancers using the Oncomine analysis tool (Figure 2). The results indicated that the mRNA expression of KMO was significantly higher in invasive ductal breast carcinoma, invasive lobular breast carcinoma, lobular breast carcinoma, invasive breast carcinoma, and ductal breast carcinoma in situ compared with matched normal tissues. Collectively, our findings revealed that the upregulation of KMO is highly correlated with breast cancer and that KMO potentially plays an important role in the tumorigenesis of breast cancers.

### 3.2. KMO Is Overexpressed in Patients with Aggressively Malignant Breast Cancers

**Figure 3:**
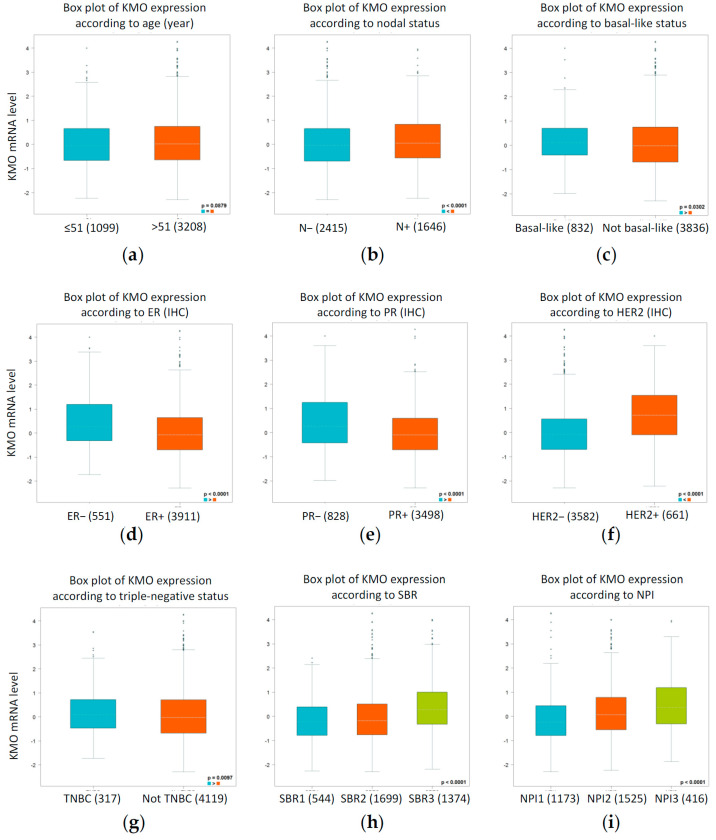
The relation between the KMO mRNA expression level and clinicopathological parameters in patients with breast cancer using the bc-GenExMiner 4.4. Association between KMO mRNA expression and (**a**) age (≤51 and ≥51 years); (**b**) nodal status (N+ vs. N−); (**c**) basal-like subtype; (**d**) ER status (ER+ vs. ER−); (**e**) PR status (PR+ vs. PR−); (**f**) HER2 status (HER2+ vs. HER2−); (**g**) triple-negative status (TNBC vs. not TNBC); (**h**) SBR grade (SBR1, SBR2 and SBR3); and (**i**) NPI grade (NPI1, NPI2 and NPI3). (ER, estrogen receptor; HER2, human epidermal growth factor receptor 2; IHC, immunohistochemistry; NPI, Nottingham prognostic index; NS, not significant; SBR, Scarff–Bloom–Richardson; TNBC, triple-negative breast cancer.).

To probe the association between different clinicopathological parameters and KMO expression in patients with breast cancer, the Breast Cancer Gene-Expression Miner (bc-GenExMiner), which evaluated the in vivo prognostic role of KMO in breast cancer, was used [27]. The expression of KMO mRNA showed no significant difference in age with a cut-off value of 51 years of age (*p* = 0.0879, Figure 3a). However, the KMO expression was significantly higher in patients with a positive nodal status (N+) than in those with a negative nodal status (N−) (*p* < 0.0001, Figure 3b). Compared with a negative basal-like status, there was a significant difference in the expression of the KMO mRNA in the basal-like status (*p* = 0.0302, Figure 3c). Additionally, the expression of the KMO mRNA was significantly different in the ER status (ER− > ER+, *p* < 0.0001, Figure 3d), PR status (PR− > PR+, *p* < 0.0001, Figure 3e), and HER2 status (HER2+ > HER2−, *p* < 0.0001, Figure 3d). Notably, patients with TNBC showed a significantly increased KMO expression compared with those in the non-TNBC group (*p* = 0.0097, Figure 3f). The analysis of the Scarff, Bloom, and Richardson grade status (SBR) criterion showed that an increased SBR level was significantly associated with an elevated KMO transcript level (SBR1 < SBR2 < SBR3, *p* < 0.0001, Figure 3h). The Nottingham prognostic index (NPI) grade showed a consistent trend (NPI1 < NPI2 < NPI3, *p* < 0.0001, Figure 3i). These clinical features were all consistent with the *TACC3*, a tumor-related gene of breast cancer [34], which indicates that KMO could serve as a potential diagnostic indicator in breast cancer as well.

### 3.3. Elevated KMO Expression Leads to Shorter Overall Survival and Relapse-Free Intervals in Patients with Breast Cancer

**Figure 4:**
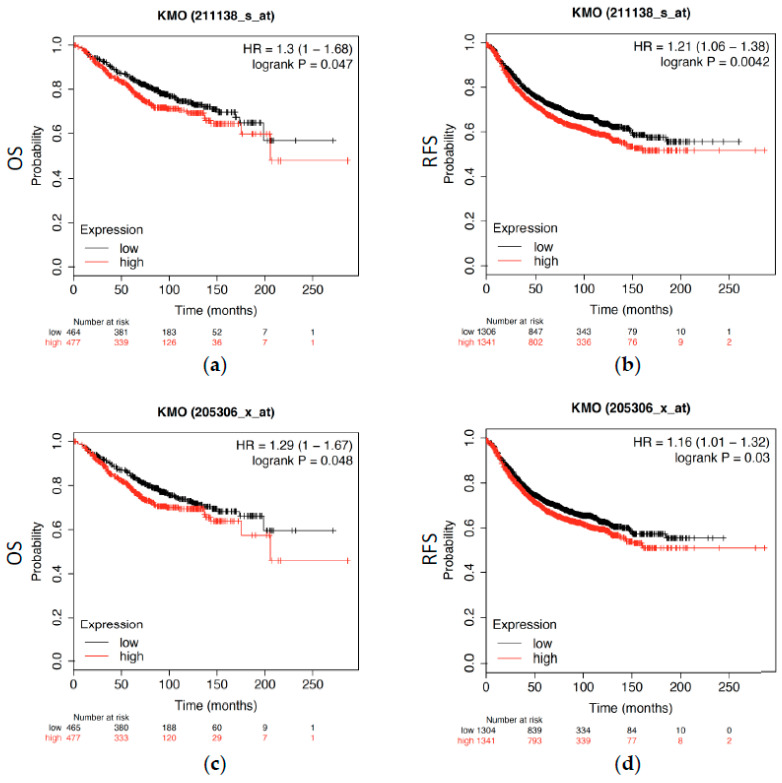
The prognostic value of KMO mRNA levels in breast cancer patients using the Kaplan–Meier plotter. Association between KMO (**a**,**b**) 211138_s_at and (**c**,**d**) 205306_x_at expression, and the OS and RFS rates in patients with breast cancer. (OS, overall survival; RFS, relapse-free survival).

To assess whether the overexpression of KMO could affect the overall survival (OS) and relapse-free survival (RFS) rates, we used the KM plotter to analyze the clinical outcomes of KMO expression. KM analysis revealed that a high KMO expression (Affymetrix microarrays, 211138_s_at and 205306_x_at; Figure 4) in breast cancer tissues was significantly associated with shorter OS (*p* < 0.05) and RFS (*p* < 0.05) rates in patients with breast cancer. Together, our findings suggest that the KMO is a tumor-related gene that participates in the tumorigenesis of breast cancer and is negatively correlated with prognosis in patients.

### 3.4. KMO-Correlated Genes Are Highly Associated with Inflammation and Potentially Facilitate Tumor Development

**Figure 5:**
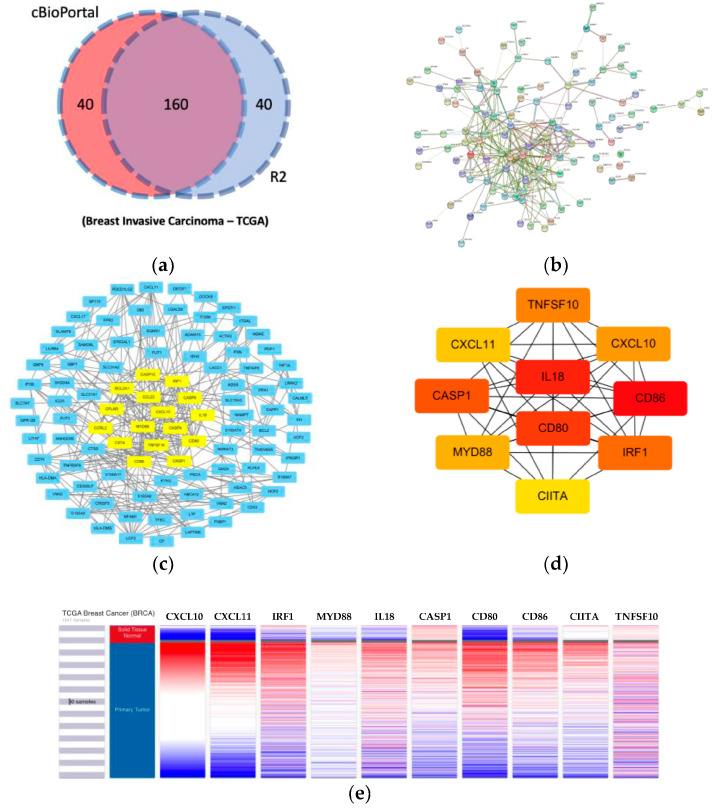
PPI network construction and hub genes analysis of KMO-correlated genes. (**a**) Venn diagram representing the intersection of the top 200 correlated genes with KMO based on the cBioPortal and R2 database and breast invasive carcinoma (TCGA, BRCA); (**b**) the protein interaction nodes of KMO-correlated genes predicted using the STRING database; (**c**) cluster analysis of KMO-correlated genes using the MCODE plugin of Cytoscape; (**d**) hub genes analyzed using the cytoHubba plugin of Cytoscape; (**e**) hierarchical clustering of hub genes constructed using the UCSC Xena online exploration tool.

In total, 200 genes correlated with KMO in breast invasive carcinoma (TCGA, Firehose Legacy) were analyzed using the cBioPortal database (Table S1, Spearman’s correlation ≥ 0.309, *p*-value ≤ 8.53 × 10^−26^). In addition, the R2 database (breast invasive carcinoma, TCGA) was used to select the top 200 genes correlated with KMO (Table S2, Correlation coefficient ≥ 0.323, *p*-value ≤ 3.10 × 10^−26^). To obtain a balance between the core genes and to avoid missing any key genes, the two databases were used to identify potential hub genes and the 160 overlapping genes were then selected as the KMO-correlated genes using Venn diagrams for further analysis (Figure 5a). To depict the interactions between these correlated genes, the 160 genes were mapped to the protein–protein interactions (PPI) from the STRING database (Figure 5b). The entire PPI network was analyzed and the most crucial module was obtained using the Cytoscape software (MCODE plugin) (Figure 5c). Furthermore, the hub genes, including *CXCL10*, *CXCL11*, *CD80*, *IRF1*, *IL18, CD86*, *CIITA*, *CASP1*, *MYD88*, and *TNFSF10*, were identified using the CytoHubba plugin via the Maximal Clique Centrality (MCC) method (Figure 5d). Notably, the majority of the hub genes were consistent with those identified in the MCODE analysis. To describe the correlation of these top 10 hub genes, they were further analyzed using the UCSC Xena. Figure 5e shows that the top 10 hub genes displayed a relatively consistent expression pattern with KMO, especially the *CXCL10* and *CXCL11* genes, which indicated that KMO possibly promotes tumor development with these correlated genes. Most of the correlated genes belonged to the chemokines and pro-inflammatory cytokines groups, which could facilitate tumor formation. For example, the binding of *CXCL10* and *CXCL11* to their corresponding receptor, CXCR3, activates the mitogen-activated protein kinase (MAPK) and phosphoinositide-3 kinase (PI3K) pathways, which supports cell motility, chemotaxis, migration, and invasion [35,36,37,38,39]. Additionally, IRF1 can interact with MyD88 to produce pro-inflammatory cytokines [40], and the pro-IL-18 can be processed into IL-18 by caspase-1, thereby promoting inflammation [41]. Collectively, these results indicated that the KMO may enable tumor progression together with its correlated genes by triggering inflammatory responses within tumors.

## 4. Discussion

Numerous therapies are now available for patients with BC; however, the high morbidity and mortality rates of BC imply that the tumorigenesis of BC needs to be further elucidated. This study aimed to analyze the role of KMO in the establishment and aggressiveness of breast cancer tumors. Moreover, the molecular mechanisms of KMO involved in enabling cancer growth were also investigated. A high level of KMO is correlated to the malignancy of breast cancers, leading to a poor prognosis in patients, which indicates that KMO could be a hub regulator and/or biomarker in breast cancers. Furthermore, the key genes correlated with KMO, such as the *MYD88*, *IRF1*, *IL-18*, and *CASP1* genes, manipulate the inflammation where many cancers arise [42].

Previous studies have also investigated the role of KMO in BC using bioinformatics tools. Huang et al. [15] firstly reported that KMO is one of the most frequently altered genes in BC and that a high level of KMO is associated with a poorer survival rate. However, the correlation between the KMO and certain clinicopathological parameters, and the synergistic effects of KMO co-expressed genes in BC patients are still under described. To the authors’ best knowledge, this study is the first to investigate the integrated characteristics of KMO and to analyze the interaction between KMO and its correlated genes in BC. KMO expression in various types of cancer, including BC, has been explored. In our previous study, the overexpression of KMO was correlated with malignancy by promoting cell proliferation and metastasis in TNBC cells [11]. Elevated KMO levels also led to a poor prognosis in patients with TNBC [15], colorectal cancer [43], and HCC [16]. Taken together, the malignant role of the KMO was reported, which aligns with our findings from using numerous bioinformatics tools. These results indicate that the KMO would serve as a useful prognosis indicator for patients with BC. 

KMO participates in tumorigenesis and it has been extensively observed playing a key role in suppressing antitumor immune responses [44,45]. A previous study showed that the genes involved in kynurenine pathways, *TDO2*, *KMO*, *KYNU*, 3*HAO*, and *QPRT*, were significantly elevated in TNBC patients [46]. This finding suggested that the activation of the kynurenine pathway is proportional to the more aggressive subtypes of breast cancer. However, previous studies focused on the expression and function of indoleamine 2,3-dioxygenase (IDO), the first step enzyme of the kynurenine pathway, but rarely described the secondary step enzyme, KMO, in tumorigenesis [47,48]. Furthermore, KMO overexpression is correlated to the enhancement of the signal transducer and activator of the transcription 3 (STAT3) and pSTAT3 [49] genes, which are related to the proliferation, survival, invasiveness, malignancy, and metastasis of tumor cells [50,51]. In this study, we found that the transcript-level expression of the KMO significantly increased in many types of cancers, such as breast cancers (Figure 1 and Figure 2). Notably, an elevated KMO is positively correlated with “relatively malignant” breast cancers, including with TNBC [4], node-positive [52], and patients with a high grade of NPI [53] (Figure 3). Moreover, a high KMO expression in breast cancers led to a worsening prognosis (Figure 4). These findings result from two possible reasons. First, the upregulation of KMO can suppress the antitumor immune responses in patients with breast cancers [44,45], and second, with an elevated KMO expression, the activation of the kynurenine pathway, via STAT3 and pSTAT3, potentially promotes the development of tumors into more aggressive phenotypes. Collectively, the current study suggests that KMO is a significant protein in the kynurenine pathway affecting cancer development.

The molecular interplays between KMO and the co-expressed genes were also investigated here, and the results revealed that they cause tumor-promoting inflammation and thereby expedite tumor progression. The hub genes which correlate to the KMO are strongly associated with inflammation, such as the *MYD88*, *IRF1*, *IL-18*, and *CASP1* genes (Figure 5). The MyD88 signaling pathway can be initiated by the toll-like receptor 7/9 and interact with the IRF1 gene to secrete pro-inflammatory cytokines [40]. In addition, pro-IL-18 can be processed into IL-18 by caspase-1, thereby promoting inflammation [41]. The *CXCL10* and *CXCL11* are also associated with KMO in regulating immune cell migration, differentiation, and activation in tumors [54]. An increased *CXCL10* and its corresponding receptor, CXCR3, are positively associated with malignant melanoma [55], ovarian carcinoma [56], multiple myeloma [57], B-cell lymphoma [58], and basal cell carcinoma [59]. A previous study also showed that ectopic KMO expression promotes metastasis in hepatocellular carcinoma [16]. Therefore, KMO and its co-expressed genes could synergistically facilitate tumor progression via enhancing chronic inflammation and triggering chemokines within the tumor microenvironment. These results possibly explain why patients with high levels of KMO usually have more progressive tumors and shorter overall survival rates. Hence, KMO has the potential to serve as a biomarker for the prediction of tumor development or grading, and it could even be designed as a therapeutic agent for immunotherapy.

KMO promotes the progression of cancers alongside its co-expressed genes and is overexpressed in patients suffering from breast cancer. This molecular characterization makes KMO a potential cancer biomarker for objectively describing the risk of clinical outcomes, such as for cancer recurrence or the reflection on therapeutic intervention [60]. However, this study had some limitations. First, only an in silico analysis was performed. Second, a large number of clinical samples, such as patient IHC stains, would be needed to support these findings, and although further evidence is needed, our data do suggest that KMO plays a vital role in human breast cancers by facilitating the proliferation, invasion, and metastasis of tumors. Heightened levels of KMO expression were found in relatively malignant breast cancers, which ultimately resulted in a poor patient prognosis. Collectively, our data reveal the tumorigenicity of KMO and provide evidence that KMO can be an effective biomarker for further studies in human breast cancers.

## 5. Conclusions

Our previous studies have reported that the expression of KMO is positively correlated with tumor progression in canine tumors and human TNBC. In this study, we have further investigated cancer clinical databases—namely, the TCGA and GTEx databases—to verify that, in addition to TNBC, KMO and its correlated genes can be used as important indicators of prognosis in different types of human BC. Wet-lab results can be used as evidence with the results from bioinformatics analyses, in which KMO is a malignant gene and a vital indicator for cancer prognosis. In summary, KMO has important clinical value in the prediction of BC development; however, further studies are needed to consider whether these biomarkers can also be used in the early diagnosis of BC.

## Data Availability

The data presented in this study are available on request from the corresponding author.

## Supplementary Materials

The following are available online at https://www.mdpi.com/article/10.3390/jpm11100948/s1: Table S1: list of top 200 genes correlated with KMO expression in the cBioPortal (*p* < 0.001); Table S2: list of top 200 genes correlated with KMO expression in R2 (*p* < 0.001).
