# Supplementary material for: Integrated Molecular Characterization to Reveal the Association between Kynurenine 3-Monooxygenase Expression and Tumorigenesis in Human Breast Cancers
Source: J Pers Med. 2021 Sep 24;11(10):948. doi: 10.3390/jpm11100948 (PMC8539700; doi:10.3390/jpm11100948)
Supplement: Supplementary file 1 [file jpm-11-00948-s001.zip › jpm-1350306-supplementary.pdf]

Supplementary Table S1. List of top 200 genes correlated with *KMO* expression in cBioPortal (p<0.001)

Correlated Gene	Cytoband	Spearman's Correlation	P-Value	q-Value
SPINK8	3p21.31	0.502850055	1.50E-71	3.04E-67
PLEKHG7	12q22	0.474785666	6.32E-63	4.61E-59
KYNU	2q22.2	0.474665207	6.85E-63	4.61E-59
C12ORF74	12q22	0.449189192	9.90E-56	5.00E-52
ABCA12	2q35	0.420624948	2.15E-48	8.67E-45
GGCT	7p14.3	0.419827028	3.36E-48	1.13E-44
S100A9	1q21.3	0.417571901	1.19E-47	3.43E-44
DEGS1	1q42.11	0.417313337	1.37E-47	3.47E-44
VNN3	6q23.2	0.414141118	7.97E-47	1.79E-43
CASP8	2q33.1	0.412854641	1.62E-46	3.26E-43
S100A8	1q21.3	0.411461954	3.47E-46	6.36E-43
PHEX	Xp22.11	0.405336724	9.51E-45	1.60E-41
ZNF599	19q13.11	-0.39869416	3.19E-43	4.96E-40
FUT3	19p13.3	0.398073299	4.41E-43	6.37E-40
TMEM86A	11p15.1	0.397664434	5.46E-43	7.35E-40
CELSR2	1p13.3	-0.395792369	1.44E-42	1.82E-39
SP110	2q37.1	0.392190013	9.22E-42	1.10E-38
CYP4Z2P	1p33	0.390830667	1.85E-41	2.07E-38
SP6	17q21.32	0.382528878	1.19E-39	1.26E-36
TC2N	14q32.12	0.382383102	1.28E-39	1.29E-36
IYD	6q25.1	0.38106288	2.45E-39	2.36E-36
CASP4	11q22.3	0.377834034	1.19E-38	1.09E-35
ASB13	10p15.1	-0.377316357	1.53E-38	1.34E-35
DAPP1	4q23	0.376552099	2.22E-38	1.86E-35
IFNGR1	6q23.3	0.376039695	2.84E-38	2.29E-35
VNN2	6q23.2	0.375301497	4.06E-38	3.15E-35
PLCH1	3q25.31	0.37409416	7.26E-38	5.42E-35
DMRTC2	19q13.2	0.37350087	9.64E-38	6.95E-35
CTSS	1q21.3	0.372643602	1.45E-37	1.01E-34
NMNAT3	3q23	-0.370138048	4.79E-37	3.22E-34
CXCL17	19q13.2	0.368910048	8.55E-37	5.57E-34
GSDMC	8q24.21	0.368691901	9.48E-37	5.98E-34
TFCP2L1	2q14.2	-0.368430985	1.07E-36	6.56E-34
NCEH1	3q26.31	0.365788558	3.70E-36	2.19E-33
NFAM1	22q13.2	0.365494896	4.24E-36	2.44E-33
RNF149	2q11.2	0.363755733	9.51E-36	5.33E-33
S100A7A	1q21.3	0.363530126	1.06E-35	5.76E-33
HDAC5	17q21.31	-0.362925938	1.40E-35	7.41E-33
LACC1	13q14.11	0.362152272	1.99E-35	1.03E-32
PRELID3A	18p11.21	-0.361908602	2.23E-35	1.13E-32
CCRL2	3p21.31	0.36166407	2.50E-35	1.23E-32
ACSL5	10q25.2	0.3613976	2.82E-35	1.36E-32
FH	1q43	0.359663152	6.24E-35	2.93E-32
S100A7	1q21.3	0.359293666	7.38E-35	3.39E-32
PGBD1	6p22.1	-0.358613318	1.01E-34	4.51E-32
PCED1B	12q13.11	0.358432191	1.09E-34	4.80E-32
FUT1	19q13.33	-0.356838922	2.25E-34	9.66E-32
LYPD4	19q13.2	0.355363117	4.37E-34	1.84E-31
CATSPERB	14q32.12	0.354027104	7.96E-34	3.28E-31

ARHGDIB	12p12.3	0.353763502	8.95E-34	3.62E-31

Correlated Gene	Cytoband	Spearman's Correlation	P-Value	q-Value
IFI30	19p13.11	0.353718044	9.14E-34	3.62E-31
SP140L	2q37.1	0.353059875	1.23E-33	4.76E-31
ST6GAL1	3q27.3	0.352974847	1.27E-33	4.85E-31
CP	3q24-q25.1	0.352816645	1.37E-33	5.11E-31
ACTR3	2q14.1	0.352712	1.43E-33	5.25E-31
DBI	2q14.2	0.352432502	1.62E-33	5.84E-31
S100A11	1q21.3	0.352374278	1.66E-33	5.89E-31
SRD5A3	4q12	0.351727557	2.22E-33	7.71E-31
SP140	2q37.1	0.351259558	2.73E-33	9.33E-31
MYD88	3p22.2	0.351070099	2.97E-33	9.98E-31
UCP2	11q13.4	0.349821467	5.15E-33	1.70E-30
LPXN	11q12.1	0.349795146	5.21E-33	1.70E-30
TNIP3	4q27	0.349316782	6.43E-33	2.06E-30
PLEKHS1	10q25.3	0.348507362	9.17E-33	2.89E-30
ACY3	11q13.2	0.348146941	1.07E-32	3.33E-30
MEGF8	19q13.2	-0.347954741	1.17E-32	3.57E-30
FNBP1	9q34.11	0.347862734	1.22E-32	3.66E-30
CASTOR3	7q22.1	-0.347111205	1.69E-32	5.01E-30
ENHO	9p13.3	-0.346893768	1.86E-32	5.43E-30
P2RY10	Xq21.1	0.345398647	3.56E-32	1.03E-29
SQOR	15q21.1	0.344675347	4.86E-32	1.38E-29
SGMS1	10q11.23	0.343936152	6.69E-32	1.88E-29
ABCC12	16q12.1	0.343608656	7.70E-32	2.13E-29
PDP1	8q22.1	0.343004587	9.99E-32	2.73E-29
SLC9A2	2q12.1	0.342869537	1.06E-31	2.85E-29
PSME4	2p16.2	0.342299075	1.35E-31	3.56E-29
KDM7A-DT	7q34	0.342291931	1.36E-31	3.56E-29
CCL22	16q21	0.342087368	1.48E-31	3.83E-29
PSCA	8q24.3	0.341928212	1.59E-31	4.05E-29
RNF114	20q13.13	0.341588126	1.83E-31	4.63E-29
SAMD9L	7q21.2	0.341351182	2.03E-31	5.06E-29
TRPV6	7q34	0.339091143	5.31E-31	1.31E-28
IDH2	15q26.1	0.338799354	6.00E-31	1.46E-28
CLCA2	1p22.3	0.33815022	7.90E-31	1.90E-28
IKBKE	1q32.1	0.337463229	1.06E-30	2.51E-28
LBP	20q11.23	0.336878659	1.35E-30	3.17E-28
SH2D4A	8p21.3	0.336121044	1.85E-30	4.30E-28
DNAJC5B	8q13.1	0.335834417	2.09E-30	4.80E-28
TNFAIP8	5q23.1	0.335417822	2.49E-30	5.64E-28
CFLAR	2q33.1	0.33519268	2.73E-30	6.13E-28
NAMPT	7q22.3	0.335047084	2.91E-30	6.44E-28
CCDC160	Xq26.2	0.33428484	3.99E-30	8.76E-28
GGT7	20q11.22	-0.334175681	4.18E-30	9.07E-28
TNFSF10	3q26.31	0.333879675	4.72E-30	1.01E-27
PLPPR3	19p13.3	-0.333290812	6.03E-30	1.28E-27
SPTSSA	14q13.1	0.333252393	6.13E-30	1.29E-27
CRISP3	6p12.3	0.332628109	7.94E-30	1.65E-27
NEURL1	10q24.33	-0.331993235	1.03E-29	2.13E-27
TFEC	7q31.2	0.331889206	1.08E-29	2.20E-27
IL18	11q23.1	0.331493819	1.27E-29	2.56E-27

Correlated Gene	Cytoband	Spearman's Correlation	P-Value	q-Value
ITGAL	16p11.2	0.331455763	1.29E-29	2.57E-27
CXCL10	4q21.1	0.331202185	1.43E-29	2.83E-27
PLEKHA8	7p14.3	0.330810152	1.68E-29	3.29E-27
BCL2A1	15q25.1	0.330096895	2.25E-29	4.37E-27
FUZ	19q13.33	-0.329950641	2.39E-29	4.58E-27
FPR3	19q13.41	0.329933935	2.41E-29	4.58E-27
SLC5A6	2p23.3	0.329774427	2.57E-29	4.84E-27
TMEM123	11q22.2	0.329159019	3.30E-29	6.17E-27
TMEM138	11q12.2	0.328687178	4.00E-29	7.41E-27
NIPAL2	8q22.2	0.328308682	4.67E-29	8.57E-27
LITAF	16p13.13	0.328045786	5.20E-29	9.45E-27
CLSTN2	3q23	-0.327856409	5.61E-29	1.01E-26
BCL2	18q21.33	-0.327645169	6.12E-29	1.09E-26
ORM2	9q32	0.326440367	9.97E-29	1.76E-26
VOPP1	7p11.2	0.325752134	1.32E-28	2.31E-26
ADSS	1q44	0.325679449	1.35E-28	2.36E-26
SLC31A2	9q32	0.325642512	1.38E-28	2.37E-26
HIF1A	14q23.2	0.325576957	1.41E-28	2.42E-26
PPA1	10q22.1	0.324590793	2.10E-28	3.56E-26
CASP1	11q22.3	0.324425723	2.24E-28	3.77E-26
SLC31A1	9q32	0.324209777	2.45E-28	4.08E-26
SLAMF8	1q23.2	0.324040226	2.62E-28	4.33E-26
LGALS9	17q11.2	0.323721914	2.97E-28	4.88E-26
ITGB6	2q24.2	0.323393842	3.39E-28	5.52E-26
LTF	3p21.31	0.323365313	3.43E-28	5.54E-26
GRAMD1A	19q13.11	-0.323304853	3.51E-28	5.63E-26
IRF1	5q31.1	0.323176757	3.70E-28	5.88E-26
ADGRG6	6q24.2	0.323157076	3.73E-28	5.88E-26
UMODL1	21q22.3	0.322896205	4.14E-28	6.47E-26
DOP1B	21q22.12	0.322603407	4.65E-28	7.22E-26
SLC7A7	14q11.2	0.322411723	5.02E-28	7.73E-26
SLC15A3	11q12.2	0.322171335	5.52E-28	8.44E-26
CXCL11	4q21.1	0.322051695	5.79E-28	8.79E-26
GBP5	1p22.2	0.321954135	6.02E-28	9.07E-26
STC2	5q35.2	-0.320440809	1.10E-27	1.64E-25
TPK1	7q35	0.32040092	1.11E-27	1.65E-25
PHLPP1	18q21.33	-0.32013869	1.24E-27	1.82E-25
STYX	14q22.1	0.318942701	1.98E-27	2.89E-25
CD53	1p13.3	0.318776026	2.11E-27	3.07E-25
MEMO1	2p22.3	0.318717748	2.16E-27	3.12E-25
PXN	12q24.23	0.318664248	2.21E-27	3.15E-25
LINC00526	18p11.31	-0.318651217	2.22E-27	3.15E-25
FBLL1	5q34	-0.318581009	2.28E-27	3.22E-25
ICOS	2q33.2	0.318542011	2.32E-27	3.25E-25
ADAM15	1q21.3	0.318374887	2.47E-27	3.44E-25
SLC12A1	15q21.1	0.317989656	2.87E-27	3.97E-25
AGBL5	2p23.3	-0.317975929	2.89E-27	3.97E-25
NABP1	2q32.3	0.317897564	2.98E-27	4.06E-25
ORM1	9q32	0.317133525	4.02E-27	5.44E-25

KLHL6	3q27.1	0.316905395	4.39E-27	5.89E-25

Correlated Gene	Cytoband	Spearman's Correlation	P-Value	q-Value
HLA-DMA	6p21.32	0.316897713	4.40E-27	5.89E-25
CD80	3q13.33	0.316837318	4.51E-27	5.99E-25
CALML5	10p15.1	0.316804563	4.57E-27	6.03E-25
APOL6	22q12.3	0.316475459	5.19E-27	6.81E-25
CD86	3q13.33	0.316393024	5.36E-27	6.98E-25
AFF3	2q11.2	-0.31601368	6.21E-27	8.04E-25
GBP1	1p22.2	0.315872799	6.56E-27	8.44E-25
CD74	5q33.1	0.315706295	7.00E-27	8.94E-25
CASP10	2q33.1	0.315382448	7.94E-27	1.01E-24
LRRK2	12q12	0.314568413	1.09E-26	1.37E-24
NAGS	17q21.31	-0.31455411	1.09E-26	1.37E-24
ZNRF2P1	7p14.3	-0.314297291	1.21E-26	1.51E-24
ARHGEF38	4q24	0.314112332	1.30E-26	1.60E-24
DNAH8	6p21.2	0.314105563	1.30E-26	1.60E-24
EPSTI1	13q14.11	0.313696397	1.52E-26	1.86E-24
DOCK8	9p24.3	0.313641023	1.56E-26	1.89E-24
NEK6	9q33.3	0.313611108	1.57E-26	1.90E-24
TNFRSF9	1p36.23	0.31336365	1.73E-26	2.08E-24
PIP	7q34	0.312932273	2.04E-26	2.44E-24
MBD1	18q21.1	-0.312781436	2.17E-26	2.57E-24
PDCD1LG2	9p24.1	0.312614116	2.31E-26	2.73E-24
CBR3	21q22.12	0.312334743	2.57E-26	3.02E-24
NCOR2	12q24.31	-0.312257731	2.65E-26	3.09E-24
SLC50A1	1q22	0.312232775	2.67E-26	3.10E-24
HTRA4	8p11.22	0.312094119	2.82E-26	3.25E-24
CD300LF	17q25.1	0.311862065	3.08E-26	3.51E-24
INAVA	1q32.1	0.311854627	3.09E-26	3.51E-24
TBCB	19q13.12	-0.311846367	3.10E-26	3.51E-24
LAPTM5	1p35.2	0.311819419	3.13E-26	3.53E-24
KDM4B	19p13.3	-0.311474854	3.57E-26	3.99E-24
XIRP1	3p22.2	0.311469486	3.58E-26	3.99E-24
NKAIN1	1p35.2	-0.311357514	3.73E-26	4.14E-24
GCH1	14q22.2	0.311244663	3.90E-26	4.30E-24
LILRB4	19q13.42	0.311094952	4.13E-26	4.53E-24
LCP2	5q35.1	0.310866297	4.50E-26	4.91E-24
CIITA	16p13.13	0.310816305	4.59E-26	4.98E-24
NCF2	1q25.3	0.310770379	4.67E-26	5.04E-24
RERG	12p12.3	-0.31068792	4.82E-26	5.18E-24
RGS12	4p16.3	-0.310674045	4.85E-26	5.18E-24
CCR8	3p22.1	0.31052155	5.13E-26	5.44E-24
ST3GAL3	1p34.1	-0.310513501	5.15E-26	5.44E-24
IGSF6	16p12.2	0.310273978	5.64E-26	5.93E-24
B2M	15q21.1	0.309889636	6.53E-26	6.83E-24
SEPTIN5	22q11.21	-0.309868115	6.58E-26	6.85E-24
UBD	6p22.1	0.309741804	6.91E-26	7.15E-24
POMGNT1	1p34.1	-0.309537605	7.46E-26	7.68E-24
TTC39B	9p22.3	0.309515129	7.53E-26	7.71E-24
HLA-DMB	6p21.32	0.309499513	7.57E-26	7.72E-24
GM2A	5q33.1	0.309345848	8.02E-26	8.14E-24
CCL20	2q36.3	0.309185297	8.53E-26	8.61E-24

Supplementary Table S2. List of top 200 genes correlated with *KMO* expression in R2 (p<0.001)

Correlated Gene	R	P-Value
SPINK8	0.514	6.60E-73
KYNU	0.493	3.30E-66
PLEKHG7	0.478	1.60E-61
S100A9	0.436	5.80E-50
TMEM86A	0.435	1.00E-49
S100A8	0.428	7.60E-48
GGCT	0.426	1.60E-47
FUT3	0.419	1.20E-45
IFNGR1	0.417	2.60E-45
CASP4	0.416	4.90E-45
PHEX	0.415	8.10E-45
ABCA12	0.412	4.90E-44
DAPP1	0.41	1.50E-43
SP140L	0.407	7.00E-43
CASP8	0.407	5.70E-43
VNN2	0.403	6.00E-42
C12orf74	0.402	8.00E-42
ASB13	-0.41	1.40E-43
CELSR2	-0.407	7.70E-43
DEGS1	0.4	2.80E-41
VNN3	0.396	1.90E-40
CTSS	0.395	3.10E-40
SP6	0.395	2.90E-40
NFAM1	0.391	2.90E-39
PLCH1	0.39	5.10E-39
TC2N	0.39	4.00E-39
ACSL5	0.389	7.20E-39
CXCL17	0.384	7.30E-38
CP	0.383	1.70E-37
IYD	0.383	1.20E-37
SRD5A3	0.382	2.70E-37
CLCA2	0.382	2.20E-37
CYP4Z2P	0.38	7.00E-37
TRPV6	0.378	1.30E-36
PDP1	0.378	1.30E-36
ST6GAL1	0.377	2.10E-36
CCL22	0.376	3.20E-36
CATSPERB	0.376	5.10E-36
SP110	0.375	5.20E-36
LBP	0.375	7.60E-36
HDAC5	-0.375	7.30E-36
TFCP2L1	-0.375	6.50E-36
PLEKHS1	0.374	1.20E-35
S100A7	0.373	1.40E-35
JHDM1D-AS1	0.373	1.80E-35
CCRL2	0.372	2.60E-35
ZNF599	-0.372	3.10E-35
SP140	0.371	3.50E-35
RNF149	0.371	4.90E-35
IFI30	0.371	4.50E-35

Correlated Gene	R	P-Value
DBI	0.371	3.90E-35
LACC1	0.37	7.00E-35
LPXN	0.37	5.80E-35
ARHGDIB	0.369	1.10E-34
ACTR3	0.368	1.60E-34
NABP1	0.367	2.70E-34
PCED1B	0.365	7.50E-34
TNFAIP8	0.365	7.50E-34
ABCC12	0.365	6.10E-34
S100A11	0.364	8.40E-34
P2RY10	0.364	1.10E-33
S100A7A	0.363	1.60E-33
DMRTC2	0.363	2.00E-33
TNIP3	0.362	2.70E-33
NAMPT	0.362	2.10E-33
CASP1	0.361	4.90E-33
ENHO	-0.361	3.90E-33
FNBP1	0.36	6.70E-33
MFSD2A	0.36	6.60E-33
SAMD9L	0.359	1.10E-32
SLAMF8	0.359	1.20E-32
UCP2	0.359	1.10E-32
GATS	-0.359	8.00E-33
FH	0.357	2.50E-32
ACY3	0.357	2.10E-32
TNFSF10	0.357	2.40E-32
FUT1	-0.357	2.10E-32
SH2D4A	0.356	4.00E-32
IL18	0.356	3.70E-32
PHF21B	-0.356	4.40E-32
LITAF	0.355	4.80E-32
SLMO1	-0.355	6.40E-32
PSCA	0.354	1.00E-31
SCPEP1	0.354	8.50E-32
SLC7A7	0.353	1.40E-31
ITGAL	0.353	1.30E-31
GSDMC	0.353	1.30E-31
HLA-DMA	0.353	1.70E-31
CFLAR	0.353	1.30E-31
ITGB6	0.352	2.10E-31
EHF	0.352	2.50E-31
PGBD1	-0.352	2.10E-31
PXN	0.351	2.80E-31
NMI	0.351	3.90E-31
CBR3	0.35	5.00E-31
MEGF8	-0.35	4.00E-31
CASP10	0.349	6.60E-31
CXCL11	0.349	6.40E-31
PDCD1LG2	0.349	6.90E-31
LGALS9	0.349	6.50E-31

Correlated Gene	R	P-Value
ICOS	0.348	1.20E-30
GRAMD2	0.348	1.30E-30
TFEC	0.348	1.20E-30
HIF1A	0.348	1.10E-30
GM2A	0.347	2.00E-30
CXCL10	0.347	1.60E-30
SQRDL	0.346	3.20E-30
ADGRG6	0.346	2.70E-30
NEURL1	-0.346	2.30E-30
GBP5	0.345	4.10E-30
CD53	0.345	4.00E-30
SLC9A2	0.345	4.00E-30
GRAMD1A	-0.345	4.20E-30
CD86	0.343	1.10E-29
CD74	0.343	8.90E-30
ADSS	0.342	1.30E-29
LY96	0.342	1.70E-29
SLC15A3	0.341	2.60E-29
SGMS1	0.341	2.50E-29
NCEH1	0.341	1.80E-29
SLC31A1	0.34	2.80E-29
CRISP3	0.34	2.80E-29
LTF	0.339	5.00E-29
NEK6	0.339	4.60E-29
IDH2	0.339	4.20E-29
MYD88	0.339	5.70E-29
C1orf106	0.339	4.80E-29
OPN3	0.338	8.30E-29
FPR3	0.337	9.90E-29
NCF2	0.337	1.20E-28
EPSTI1	0.336	2.00E-28
TMEM123	0.336	1.60E-28
LAPTM5	0.336	1.80E-28
DNAJC5B	0.336	1.70E-28
ADAM15	0.336	1.70E-28
CIITA	0.336	1.80E-28
BCL2A1	0.335	2.30E-28
PLEK	0.335	2.20E-28
DOCK8	0.335	2.30E-28
OLR1	0.334	4.40E-28
LILRB4	0.334	3.40E-28
TMEM138	0.334	4.50E-28
HLA-DRA	0.333	6.80E-28
HAVCR2	0.333	6.30E-28
SLC31A2	0.333	5.70E-28
PHGR1	-0.333	5.20E-28
NMNAT3	-0.333	6.40E-28
TPK1	0.332	9.30E-28
CCR5	0.332	7.40E-28
ATP13A5	0.332	8.70E-28

Correlated Gene	R	P-Value
APOL6	0.332	8.50E-28
KLHL6	0.332	7.30E-28
HTRA4	0.332	9.10E-28
TNFRSF9	0.332	7.40E-28
SPTSSA	0.332	9.60E-28
IL2RG	0.331	1.20E-27
LRRK2	0.331	1.30E-27
HLA-DMB	0.331	1.50E-27
FYB	0.331	1.40E-27
CD2	0.33	2.30E-27
SLC12A1	0.33	2.20E-27
STK17B	0.329	2.40E-27
PTPN22	0.329	3.30E-27
CD80	0.329	2.80E-27
BCL2	-0.329	2.40E-27
TTC39B	0.328	4.50E-27
IRF1	0.328	4.20E-27
APOC1	0.328	3.70E-27
CST9	-0.328	4.10E-27
KLRD1	0.327	6.10E-27
CD68	0.327	6.90E-27
ACTB	0.327	5.00E-27
CXCL9	0.327	6.80E-27
SWAP70	0.327	6.10E-27
SYTL3	0.327	5.80E-27
GGT7	-0.327	5.60E-27
LCP2	0.326	9.00E-27
LAIR1	0.326	7.50E-27
ADAM8	0.326	8.90E-27
EVI2B	0.326	9.30E-27
GPR137B	0.326	7.50E-27
SDC1	0.326	9.10E-27
LPPR3	-0.326	9.30E-27
GBP1	0.325	1.10E-26
ICAM1	0.325	1.40E-26
CD300LF	0.325	1.50E-26
NPL	0.325	1.30E-26
POMGNT1	-0.325	1.30E-26
XIRP1	0.324	1.80E-26
PPA1	0.324	2.00E-26
ALDH2	0.324	2.20E-26
C2CD4A	0.324	1.80E-26
CCDC160	0.324	1.90E-26
OAS2	0.323	3.00E-26
UBD	0.323	2.90E-26
RNF130	0.323	2.90E-26
ARHGEF38	0.323	3.30E-26
CALML5	0.323	2.60E-26
IKBKE	0.323	2.60E-26

SNX20	0.323	3.10E-26
